# The effect of presymptomatic hypertension in posterior reversible encephalopathy syndrome

**DOI:** 10.1002/brb3.1061

**Published:** 2018-07-11

**Authors:** Moon Kyu Lee, Yang‐Je Cho, Seung‐Koo Lee, Sang Ku Jung, Kyoung Heo

**Affiliations:** ^1^ Department of Neurology Gangneung Asan Hospital Gangneung Korea; ^2^ Department of Neurology Yonsei University College of Medicine Seoul Korea; ^3^ Department of Radiology Yonsei University College of Medicine Seoul Korea; ^4^ Department of Emergency Medicine Gangneung Asan Hospital Gangneung Korea

**Keywords:** blood pressure, lesion scoring point, magnetic resonance imaging, posterior reversible encephalopathy syndrome

## Abstract

**Objective:**

The effect of blood pressure (BP) on the lesion distribution of posterior reversible encephalopathy syndrome (PRES) is controversial. The aim of this study was to identify the relationship between brain lesion distribution patterns and BP.

**Methods:**

Sixty‐five patients with PRES were selected from the database. Data regarding brain MRI findings, clinical symptoms, medical conditions, and BP at the presymptomatic period (24 hr before the symptom onset) and at the symptom onset were collected. The brain lesion distribution degree was numerically calculated (lesion scoring point [LSP]) and compared with BP and medical conditions.

**Results:**

Mean onset‐MAP was higher than mean pre‐MAP. Pre‐MAP correlated with onset‐MAP. The LSP was significantly correlated with pre‐MAP (*p *=* *0.009, correlation coefficient [cc] = 0.323), whereas no significant correlation was found between LSP and onset‐MAP (*p *=* *0.159, cc = 0.177). Similarly, when patients were grouped by mean MAP values, LSP was significantly higher in the patients with high MAP at the presymptomatic period (*p *=* *0.004), whereas no difference was found in the LSP value between patients with low MAP and high MAP at the symptom onset (*p *=* *0.272).

**Conclusion:**

The patient with PRES who has relatively higher BP in the presymptomatic period would be more likely to have wider lesion distribution than the patient with lower BP. BP elevation during presymptomatic period may be a heralding sign of impending PRES and a factor affecting the severity of PRES although BP was not investigated at earlier time points.

## 1 INTRODUCTION

Posterior reversible encephalopathy syndrome (PRES) is a clinico‐radiological syndrome (Lee, Wijdicks, Manno, & Rabinstein, 2008). Clinical symptoms are headache, visual disturbance, mental alteration, and seizure. The characteristic radiologic finding is the vasogenic edema in the posterior part of the brain (Bartynski, 2008a,2008b). Diverse brain imaging patterns are currently reported with the advances of the imaging techniques (McKinney, Jagadeesan, & Truwit, 2013). The elimination or correction of the predisposing factors usually ensures the favorable outcomes. The mechanism behind the brain edema development is controversial. The failure of auto‐regulation caused by severe hypertension or direct toxic insult to endothelium is the most popular theory for pathophysiology (Bartynski, 2008b; Dinsdale, 1983; Schwartz et al., 1995; Strandgaard, Olesen, Skinhoj, & Lassen, 1973). One of the unsettled questions about PRES is the correlation between the brain lesion distribution patterns and blood pressure (BP). The aim of this study is to identify the relationship between brain lesion distribution patterns and BP.

## 2 METHODS

### 2.1 Data collection

We investigated patients with PRES who were admitted to Severance Hospital between January 2001 and December 2014. All patients had been admitted already at least 24 hr before the symptom onset. Diagnosis of PRES was based on clinical features (predisposing conditions, headache, seizures, mental alteration, and visual disturbances); multifocal lesions on MRI, mainly suggesting vasogenic edema; clinical recovery; and when available, reversibility of MRI lesions. Clinical information such as past medical histories, comorbid illnesses, brain magnetic resonance imaging (MRI), BP measured 1 day before the symptom onset (pre‐BP), and BP checked (onset‐BP) at symptom onset (mental alteration, seizure, severe headache, or visual disturbances) was collected. BP had been measured every at least 8 hr. BP measured between 24 and 32 hr before the symptom onset was regarded as BP 1 day before the symptom onset. Mean arterial pressure (MAP) was calculated using the formula: Mean arterial pressure = 2/3 diastolic BP + 1/3 systolic BP.

Cases with unclear symptom onset, without BP documentation, without brain MRI, with pre‐existing neurologic deficits, or with a history of seizure disorder were excluded. As a result, 65 patients were selected. This study was approved by the Institutional Review Board of Severance Hospital.

### 2.2 Brain imaging and brain lesion distribution

Brain MRI using either 1.5‐T (Signa Horizon, GE Medical System, Milwaukee, WI, or Gyroscan Intera, Philips Medical Systems, Best, The Netherlands) or 3.0‐T scanner (Achieva, Philips Medical Systems, Best, The Netherlands), including conventional spin‐echo T_1_‐weighted axial, T_2_‐weighted axial, and fluid‐attenuated inversion recovery axial sequences, were commonly reviewed in all the patients. The mean time to the MRI evaluation was 1.6 days (range, 0–6 days).

**Figure 1:**
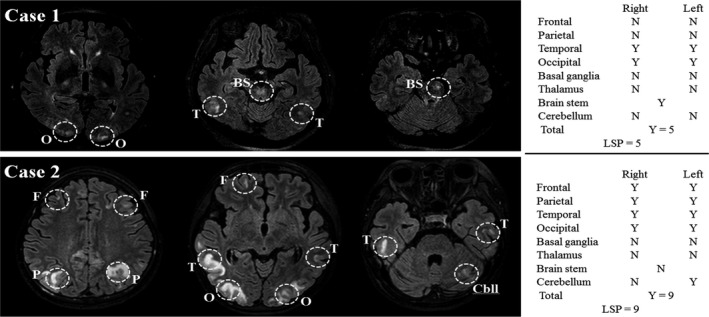
The lesion scoring method. Two sample cases are illustrated. O, occipital lobe; T, temporal lobe; BS, brain stem; F, frontal lobe; P, parietal lobe; Cbll, cerebellum; N, no; Y, yes; LSP, lesion scoring point

The lesion distribution of the PRES was determined by one experienced neuro‐radiologist (SK Lee). Affected brain regions were tabulated as frontal, parietal, occipital, temporal lobes, basal ganglia (BG), thalamus, brain stem (BS), and cerebellum (Cbll). Each brain region was scored as one point, and then the scores from the brain regions were added up (lesion scoring point, LSP) in each patient (Figure 1).

### 2.3 Statistical analysis

Statistical analysis was performed using SPSS (version 20) for Windows (IBM Corp., Armonk, NY, USA). The Chi‐square, Mann–Whitney *U*, Spearman's correlation, and Kruskal–Wallis tests were used to determine the statistical significance (*p *<* *0.05).

## 3 RESULTS

### 3.1 Patients profiles, clinical symptoms, and prognosis

All patients were in‐hospital onset PRES cases. Forty‐eight patients (73.8%) of 65 were female. The mean age was 40.0 years (median, 36 years; range, 11–90). Underlying medical conditions were as follows: chemotherapy‐immunosuppression (*n* = 26), renal failure (*n* = 22), preeclampsia‐eclampsia (*n* = 10), autoimmune disease (*n* = 9), isolated uncontrolled hypertension (*n* = 4), and others (*n* = 6; transfusion [*n* = 2], acute intermittent porphyria [*n* = 2], burn [*n* = 1], and pancreatitis [*n* = 1]) (Table 1). Multiple medical conditions were found in 12 patients of 65. Visual disturbances including blurred vision, hemianopsia, hallucination, and cortical blindness were present in 31 patients (47.7%), headache was present in 42 (64.6%), seizure was present in 51 (78.5%), limb weakness was present in 7 (10.8%), and mental alteration was found in 36 (55.4%) of 65 patients.

**Table 1:** Patient profiles

Number of patients (male/female)	65 (17/48)
Age (mean, (median, range))	40.0 (36.0, 11–90)
Medical conditions (*n*)	Chemotherapy‐immunosuppression (26)
	Renal failure (22)
	Preeclampsia‐eclampsia (10)
	Autoimmune disease (9)
	Hypertension (4)
	Others (6)

### 3.2 Brain lesion distribution

Cerebral cortical or subcortical lesions were found in 63 (96.9%) of the 65 patients. Two (3.1%) of the 65 patients did not have cerebral cortical or subcortical lesions. These individuals had lesions in deep nuclei or BS. The cerebral lesion locations were occipital in 56 (86.2%), parietal in 54 (83.1%), frontal in 44 (67.7%), and temporal in 38 (58.5%) of the 65 patients. BG, thalamus, BS, and Cbll lesions were identified in 13 (20.0%), 12 (18.5%), 8 (12.3%), and 17 (26.2%) of 65 patients. There was no significant relationship between the medical condition and the specific brain region preference except for the BG involvement in patients with preeclampsia‐eclampsia. Five of 10 patients with preeclampsia‐eclampsia had lesions in BG (*p *=* *0.010).

### 3.3 Relationship among lesion distribution, BP, and etiology

**Figure 2:**
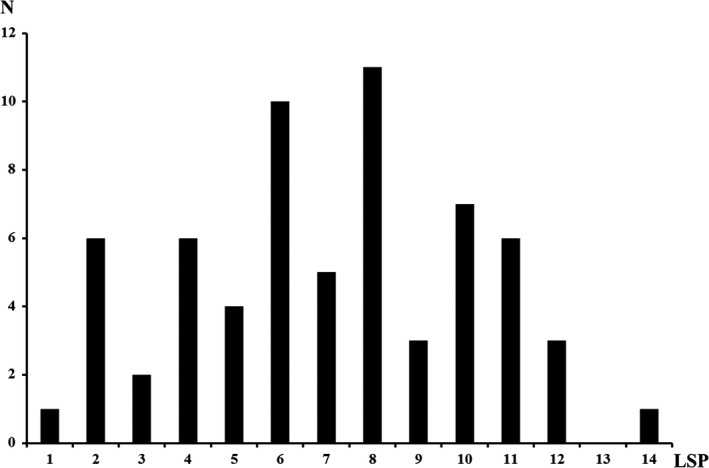
The distribution of lesion scoring point. *N*, number; LSP, lesion scoring point

The mean time of BP measurement before the symptom onset was 27.2 ± 2.5 hr (range, 24–31 hr). The mean MAP was 99.7 ± 13.8 mmHg (range, 73.3–133.3 mmHg) at the presymptomatic period and 133.0 ± 21.2 mmHg (range, 81.3–183.3 mmHg) at the symptom onset in the total patients. The onset‐MAP was significantly higher than pre‐MAP (*p *<* *0.001). In the brain lesion scoring, the mean LSP was 7.1 ± 3.1 (range, 1–14) in the total patients (Figure 2).

In the correlation analysis of BP and scoring point, LSP correlated with pre‐MAP (*p *=* *0.009, correlation coefficient [cc] = 0.323). Pre‐MAP correlated with onset‐MAP (*p *<* *0.001, cc = 0.424). No significant correlation was found between LSP and onset‐MAP (*p *=* *0.159, cc = 0.177) (Table 2).

**Table 2:** The correlation between scoring point and blood pressure

	Pre‐MAP	Onset‐MAP	LSP
Pre‐MAP		*p *<* *0.001 (cc = 0.424)	*p *=* *0.009 (cc = 0.323)
Onset‐MAP	*p *<* *0.001 (cc = 0.424)		*p *=* *0.159 (cc = 0.177)
LSP	*p *=* *0.009 (cc = 0.323)	*p *=* *0.159 (cc = 0.177)	

Note

MAP, mean arterial pressure; LSP, lesion scoring point; cc, correlation coefficient.

Patients were grouped by mean MAP values as follows; (a) low (baseline MAP < 99.7 mmHg, *n* = 28) and (b) high (baseline MAP ≥ 99.7 mmHg, *n* = 37) in the presymptomatic period, and (a) low (onset MAP < 133 mmHg, *n* = 26) and (b) high (onset MAP ≥ 133 mmHg, *n* = 39) in the symptom onset period respectively. LSP was significantly higher in the high MAP group than low MAP group in the presymptomatic period (*p *=* *0.004). No statistical difference was found in the LSP value between low MAP and high MAP groups at the symptom onset (*p *=* *0.272).

Patients were classified by underlying medical conditions and the mean pre‐MAP, onset‐MAP, and LSP were calculated. There were no significant differences in pre‐MAP, onset‐MAP, and LSP according to medical conditions (Table 3).

**Table 3:** The blood pressure profiles and scoring points

Medical conditions	Pre‐MAP, mmHg	Onset‐MAP, mmHg	LSP
Total (*n* = 65)	99.7 ± 13.8	133.0 ± 21.2	7.1 ± 3.1
CTx‐IS (*n* = 29)	96.8 ± 12.8	128.8 ± 22.3	7.0 ± 3.2
Renal failure (*n* = 22)	102.2 ± 16.3	138.9 ± 21.9	7.5 ± 3.4
Preeclampsia‐eclampsia (*n* = 10)	100.2 ± 11.7	127.3 ± 12.4	7.6 ± 2.5
Autoimmune disease (*n* = 9)	96.6 ± 11.7	129.8 ± 18.2	7.4 ± 2.4
Hypertension (*n* = 4)	116.8 ± 11.6	140.0 ± 11.5	4.8 ± 2.5
Others (*n* = 6)	93.2 ± 2.2	129.0 ± 25.2	4.3 ± 2.6

Note

CTx‐IS, Chemotherapy‐immunosuppression; LSP, lesion scoring point.

## 4 DISCUSSION

In this study, we assumed an evolution period before the toxic symptom onset. We collected the BP profiles in the presymptomatic and onset periods, and compared lesion distribution with BPs. As a result, LSP correlated with pre‐MAP although pre‐MAP did with onset‐MAP. Patients with higher BP profiles appeared to have increased chance of wider lesion distribution than patients with lower BP profiles in the presymptomatic period.

There have been several studies concerning the relationship between BP at symptom onset, MRI findings, and clinical characteristics. However, their results were incongruent. Bartynski and Boardman found four types of cerebral edema pattern which was classified by main edema lesion location and symmetricity between two cerebral hemispheres. No significant association between MAP at toxicity and imaging patterns, and MAP and underlying medical conditions was found (Bartynski & Boardman, 2007). Later, they assessed the extent and severity of hemispheric cortex‐white matter edema by visual grading method. Hemispheric edema was greater in normotensive patients than in those with moderate to severe hypertension (Bartynski & Boardman, 2008). In a study reported in 2009, authors also used a visual grading method to measure the extent of the abnormal signal. In the comparison of the hypertensive group and normotensive group at symptom onset, no significant differences in the extent of disease and the number of affected brain regions between two groups were found. The number of affected brain regions was significantly higher in patients with eclampsia, and the basal ganglia region was more frequently involved in these patients (Mueller‐Mang et al., 2009). Liman et al. enrolled 96 patients with PRES and analyzed the edema severity, lesion distribution, and BP profiles on symptom onset period. Lesion distribution was not significantly correlated with the type of toxic association. There was a significant difference in MAP between toxic associations. Onset‐MAP was higher in infection, eclampsia, and autoimmune disorders and lower in chemotherapy and immunosuppression related PRES cases. There was a trend for higher edema grades to be associated with higher systolic BP values. High MAPs were associated with incomplete lesion resolution on follow‐up MRI (Liman, Bohner, Heuschmann, Endres, & Siebert, 2012). This result implies that there would be a difference in pathophysiological processes which lead to PRES in regard to etiologies and BP may affect the course of PRES.

In a study published in 2012, authors investigated the presymptomatic BP changes in 25 PRES cases and compared with those of the controls. They recorded the BP changes over a 48‐hr window before the PRES symptom onset and calculated the degree of BP fluctuation by mathematical formula. The BP fluctuations were not more common in PRES cases than in the controls despite of significantly increased BP over the 48‐hr window. It was suggested that PRES cannot be explained solely on the basis of severe acute hypertension or sudden BP surges or fluctuation (Rabinstein et al., 2012). Although their study did not directly investigate the relationship between BP at the presymptomatic period and at symptom onset within the PRES cases, their result seems to be contrary to our result showing the significant BP elevation at the symptom onset compared with the presymptomatic period.

To our knowledge, the relationship between PRES severity and pre‐BP has not been investigated. It is interesting that LSP as a marker of PRES severity was explained better by pre‐BP rather than onset‐BP. In the Doppler study for pre‐eclampsia patients who were neurologically normal, their autoregulation indices were lower than those of healthy controls, suggesting dysfunctional autoregulation during presymptomatic period (van Veen et al., 2013). Therefore, we presume that higher BP during presymptomatic period may make worse the impairment of cerebral autoregulation and may lead to more severe PRES. BP elevation during presymptomatic period might be a heralding sign of impending PRES or an important factor affecting the severity of PRES although BP was not investigated at earlier time points. Prompt control of a rising BP would be required for the prevention of PRES in patients with risk factors for PRES.

Our study has limitations. First, there may be limitations in the detection of clinical symptoms and the estimation of onset time because of a retrospective analysis depending on the accuracy of medical records. Second, the severity of edematous lesion was not evaluated in our study. Only the lesion distribution could not reflect lesion severity appropriately. Higher pre‐BP may be more likely associated with the number of affected brain regions rather than with disease severity. Third, the required evolution period for the PRES generation may differ between individuals. Thus, the information about BP at multiple time points before the toxic symptom onset is required to investigate the preclinical hemodynamic changes of PRES. Fourth, this study included only hospitalized patients, which may produce selection bias. Also, a small number of patients prevent to reveal the relationship between etiologies and lesion distribution patterns. Above all, BP is not the sole controlling factor for cerebral autoregulation. Considering the above limitations, a cautious interpretation of the study results is needed.

## 5 CONCLUSION

This study may suggest that the patients with PRES who had higher BP in the presymptomatic period might be more likely to show wider spatial distribution of edematous lesions.

## CONFLICT OF INTEREST

The authors declare no conflict of interests.
